# Airway Epithelial Cells Generate Pro-inflammatory Tenascin-C and Small Extracellular Vesicles in Response to TLR3 Stimuli and Rhinovirus Infection

**DOI:** 10.3389/fimmu.2019.01987

**Published:** 2019-08-21

**Authors:** Jake T. Mills, Anja Schwenzer, Elizabeth K. Marsh, Michael R. Edwards, Ian Sabroe, Kim S. Midwood, Lisa C. Parker

**Affiliations:** ^1^Department of Infection, Immunity and Cardiovascular Disease, School of Medicine, Dentistry and Health, University of Sheffield, Sheffield, United Kingdom; ^2^Faculty of Biological Sciences, Astbury Centre for Structural Molecular Biology, School of Molecular and Cellular Biology, University of Leeds, Leeds, United Kingdom; ^3^Nuffield Department of Orthopaedics, Rheumatology and Musculoskeletal Sciences, Kennedy Institute of Rheumatology, University of Oxford, Oxford, United Kingdom; ^4^School of Human Sciences, College of Life and Natural Sciences, University of Derby, Derby, United Kingdom; ^5^Department of Respiratory Medicine, National Heart and Lung Institute, Imperial College London, London, United Kingdom

**Keywords:** human rhinovirus, asthma exacerbations, tenascin-C, exosomes, extracellular matrix protein, inflammation

## Abstract

Viral infections are a common cause of asthma exacerbations, with human rhinoviruses (RV) the most common trigger. RV signals through a number of different receptors, including toll-like receptor (TLR)3. Tenascin-C (TN-C) is an immunomodulatory extracellular matrix protein present in high quantities in the airway of people with asthma, and expression is also upregulated in nasal lavage fluid in response to RV infection. Respiratory viral infection has been demonstrated to induce the release of small extracellular vesicles (sEV) such as exosomes, whilst exosomal cargo can also be modified in the bronchoalveolar lavage fluid of people with asthma. These sEVs may potentiate airway inflammation and regulate the immune response to infection. This study characterizes the relationship between RV infection of bronchial epithelial cells and the release of TN-C, and the release of sEVs following stimulation with the TLR3 agonist and synthetic viral mimic, poly(I:C), as well as the function of the released protein/vesicles. The BEAS-2B airway epithelial cell line and primary human bronchial epithelial cells (PBECs) from asthmatic and non-asthmatic donors were infected with RV or treated with poly(I:C). TN-C expression, release and localization to sEVs was quantified. TN-C expression was also assessed following intra-nasal challenge of C57BL/6 mice with poly(I:C). BEAS-2B cells and macrophages were subsequently challenged with TN-C, or with sEVs generated from BEAS-2B cells pre-treated with siRNA targeted to TN-C or control. The results revealed that poly(I:C) stimulation induced TN-C release *in vivo*, whilst both poly(I:C) stimulation and RV infection promoted release *in vitro*, with elevated TN-C release from PBECs obtained from people with asthma. Poly(I:C) also induced the release of TN-C-rich sEVs from BEAS-2B cells. TN-C, and sEVs from poly(I:C) challenged cells, induced cytokine synthesis in macrophages and BEAS-2B cells, whilst sEVs from control cells did not. Moreover, sEVs with ~75% reduced TN-C content did not alter the capacity of sEVs to induce inflammation. This study identifies two novel components of the inflammatory pathway that regulates the immune response following RV infection and TLR3 stimulation, highlighting TN-C release and pro-inflammatory sEVs in the airway as relevant to the biology of virally induced exacerbations of asthma.

## Introduction

Asthma is a chronic disease characterized by airway inflammation, remodeling, and airway hyperresponsiveness (AHR) (1). Around 5–10% of those with the disease have severe asthma with poorly controlled symptoms (2), and exacerbations are an acute, frequently occurring, and potentially severe manifestation of this illness (3). One of the main causes of asthma exacerbations are respiratory viruses, with the most common viruses responsible being human rhinoviruses (RV) (4).

RV are single-stranded RNA, non-enveloped viruses, which are members of the *Picornaviridae* family and encompass around 160 serotypes. These viruses are classified either by the A-B-C classification system (based on the similarity in the RNA sequences of the viral protein 1), whereas the major-minor-C classification system is based on the entry receptor used by the virus to enter the cell (5–7). Major serotypes bind to intracellular adhesion molecule 1 (ICAM-1) on the cell surface, minor serotypes use various low density lipoprotein receptors (LDLRs) (6) and RV-C was identified in 2006 and uses cadherin-related family member 3 (CDHR3) for binding and replication (7).

Bronchial epithelial cells are the principle site of RV binding and replication, and RV are recognized by pattern recognition receptors (PRRs) including toll-like receptors (TLR)2 and TLR3, and the Retinoic acid-inducible gene-I (RIG-I)-like receptors (RLRs) melanoma differentiation-associated-protein 5 (MDA5) and retinoic acid-inducible gene (RIG-I) (8, 9). TLR and RLR activation induces interferon regulatory factor (IRF), mitogen-activated protein kinase (MAPK) and NF-κB signaling, leading to cytokine and interferon production (10, 11). Whilst this inflammation is typically readily resolved, RV infection can lead to an exaggerated response in people with asthma, resulting in excessive cytokine release and mucus hypersecretion that are characteristic of asthma exacerbations (12).

RV can promote the deposition of extracellular matrix (ECM) proteins, with tenascin-C (TN-C) mRNA expression enhanced in nasal cells following infection (13). TN-C is composed of four main domains: the tenascin assembly (TA) domain, epidermal growth factor (EGF)-like repeats, fibronectin type III (FNIII)-like repeats and fibrinogen globe-like (FBG-C) domain (14). It can range in size from 180 to 330 kDa, and expression is low in the healthy adult airway but is increased in the basement membrane of people with asthma (15). TN-C expression correlates with asthma severity in humans (15), AHR in mouse models of asthma is reduced in TN-C KO mice (16), and a single nucleotide polymorphism (SNP) in the structure of TN-C associates with adult bronchial asthma (17). TN-C is a key driver of chronic inflammation in a number of different pathologies [summarized in (18)] through both FBG-C-TLR4 and FNIII-integrin interactions. This is well-established in models of rheumatoid arthritis (RA), with the FBG-C domain interacting with TLR4 receptors on the surface of synovial fibroblasts and macrophages (14, 19). However, despite evident roles for TN-C in asthma biology, the expression of TN-C in bronchial epithelial cells and the role of TN-C in RV-induced inflammation have not been studied.

In this study we investigated bronchial epithelial cell TN-C expression and release following RV infection, and determined the function of the protein. We observed release of TN-C upon infection, and established that purified recombinant FBG-C had the ability to induce inflammatory cytokine release in bronchial epithelial cells and macrophages. Surprisingly, a large proportion of TN-C was associated with small extracellular vesicles (sEV), which have previously been implicated in asthmatic airway inflammation, and viral challenge increased the concentration of overall sEV release. sEVs from virally stimulated cells had the ability to induce inflammatory and antiviral cytokine production in bronchial epithelial cells, whilst sEVs from control cells did not. Finally, sEV induced inflammation was determined to be independent of TN-C. Thus, this study identifies TN-C and sEVs as two novel drivers of the airway inflammation that underpins asthma pathogenesis, and therefore may be potential future therapeutic targets to help control RV-induced asthma exacerbations.

## Materials and Methods

### Viral Culture

RV minor serotype 1B (RV-1B) and major serotype 16 (RV-16) were obtained from ATCC (LGC Standards, Teddington, UK) and viral stocks generated by infecting HeLa Ohio cells (ATTC) as previously described (20). The cytopathic effect was then determined and the multiplicity of infection (MOI) calculated.

### Cell Culture

The BEAS-2B epithelial cell line, and primary bronchial epithelial cells (PBECs) isolated from healthy humans, were purchased from ATCC and Promocell (Heidelberg, Germany) and cells were maintained as described (21, 22). PBECs were also obtained during bronchoscopy from adult (18–55 years old) non-atopic non-asthmatic controls (NANA) and patients with atopic asthma (AA) (23), with written informed consent, in accordance with the Declaration of Helsinki and a protocol approved by London Bridge Research Ethics committee (reference number 12/LO/1278), and maintained in the same way as the purchased cells. The AA subjects had a prior clinical diagnosis of asthma, scored > 0.75 on an asthma control questionnaire, had a histamine PC_20_ of <8 μg/ml, and atopy was confirmed by a positive skin prick test to timothy grass pollen (in a panel of 10 aeroallergens) (23). NANA subjects had a histamine PC_20_ of >8 μg/ml (23). Peripheral blood mononuclear cells (PBMCs) were isolated from peripheral venous blood of healthy volunteers (21), with written informed consent, in accordance with a protocol approved by South Sheffield Local Research Ethics Committee (reference number: 05/Q2305/4) and differentiated into monocyte-derived macrophages using a previously established method (14).

### Cell Stimulation and Infection

Cells were seeded in 6, 12 or 96 well plates, grown to confluency (80%) and placed in supplement free media overnight. For stimulation experiments, cells were stimulated with 25 μg/ml polyinosinic:polycytidylic acid (poly([I:C]) (Invitrogen, Paisley, UK), 10 μg/ml gardiquimod (Invitrogen) or 100 ng/ml or lipopolysaccharide (LPS) serotype 0111:B4 (Sigma-Aldrich) or EH100 (Enzo, Exeter, UK). For infection experiments, BEAS-2B cells or PBECs were infected with RV-1B and RV-16 (ATCC) for the indicated times at optimized MOIs (10). For TN-C stimulation experiments, recombinant FBG-C protein was expressed and purified as described (14, 24), before being added to cells at the indicated concentrations for 24 h. For small extracellular vesicle (sEV) stimulation experiments, isolated sEVs (see Supplementary Methods) were added to at the indicated concentrations for 24 h. Cell free supernatants, mRNA and/or protein lysates were then harvested and stored appropriately.

### Murine Model

This study was carried out in accordance with the principles of the Basel Declaration and recommendations of Animal (Scientific Procedures) Act 1986, United Kingdom Home Office. The protocol was approved by the animal welfare and ethical review body at the University of Sheffield, and work was carried out under project license code 40/3726 and establishment license code 50/2509. Under 4% isofluorane (Abbott Laboratories Illinois, USA), C57BL/6 adult mice (see Supplementary Methods for more information) were intranasally stimulated with 50 μl PBS (Oxoid, ThermoFisher, Basingstoke, UK) or 50 μl PBS containing 100 μg poly(I:C). The mice were sacrificed and bronchoalveolar lavage fluid (BALF) collected as previously described (25).

### Western Blot

Where required, cell lysates were prepared as previously established (21). For supernatant samples, 4 × SDS loading buffer was added and samples were analyzed following the same protocol. Cell-lysate western blot samples were probed for human TN-C (N-Terminal, mab1908, Merck Millipore, California, USA), Histidine-Tag (H1029, Sigma-Aldrich), and β-actin (A5316, Sigma-Aldrich), with TN-C expression normalized to β-actin, whilst supernatant samples were analyzed for TN-C only. Mouse BALF was concentrated by trichloroacetic acid (TCA, Sigma-Aldrich) precipitation prior to analysis and TN-C expression determined using mouse TN-C antibody (N-Terminal, T3413, Sigma-Aldrich). sEV isolates were analyzed for sEV-enriched proteins cluster of differentiation 9 (CD9, sc-13118, Santa-Cruz Biotechnology, Dallas, USA) and flotillin-1 (ab13493, Abcam, Cambridge, UK), with the negative control glucose regulated protein 94 (GRP94, ab7291, Abcam) utilized to confirm the lack of cellular protein contamination. Densitometrical analysis was performed using ImageJ software (Version 1.5i; NIH). Due to multiple variants of TN-C being expressed, the dominantly expressed band was measured for each experiment.

### ELISA

Cell-free supernatants were collected and quantified for C-X-C Motif Ligand 8 (CXCL8), CCL5, and IL-5 (R&D, Minneapolis, USA) and TN-C N-Terminal Kit (Cloud Corp, Texas, USA) using matched Ab pairs by enzyme-linked immunosorbent assay (ELISA), following the manufacturer's instructions. Minimum detection levels (all pg/ml) were CXCL8: 78.125, TN-C: 125, IL-5: 156.25, and CCL5: 156.25.

### MTT Assay

The 3-(4,5-Dimethylthiazol-2-yl)-2,5-Diphenyltetrazolium Bromide (MTT) assay assessed nicotinamide adenine dinucleotide phosphate (NADPH) activity as a measure of cell metabolic activity and thus viability, and was performed as previously described (26).

### Nanoparticle Tracking Analysis

The size and concentration of the isolated EVs were analyzed by the ZetaView® Nanoparticle Tracking Analyser (Particle Metrix, Dusseldorf, Germany). Each sample was diluted in filtered PBS (1:30–1:50) to reach the optimum detection limit in the machine and measured three times.

### Statistics

Data were analyzed and presented via GraphPad Prism v7.0 (California, USA) as mean ± SEM of at least three independent experiments, with each replicate carried out on a separate BEAS-2B cell passage or independent PBEC donor, please see figure legends for specific experimental replicate numbers. Statistical tests performed are also detailed within the figure legends with significant differences indicated by *p < 0.05; **p < 0.01; ***p < 0.001; ****p < 0.0001.

## Results

### RV Infection Induces TN-C Release *in vitro* in PBECs, With Release Greater in PBECs Obtained From People With Asthma, Whilst the Viral Mimic Poly(I:C) Induces TN-C Release Into the Murine Airway

**Figure 1:**
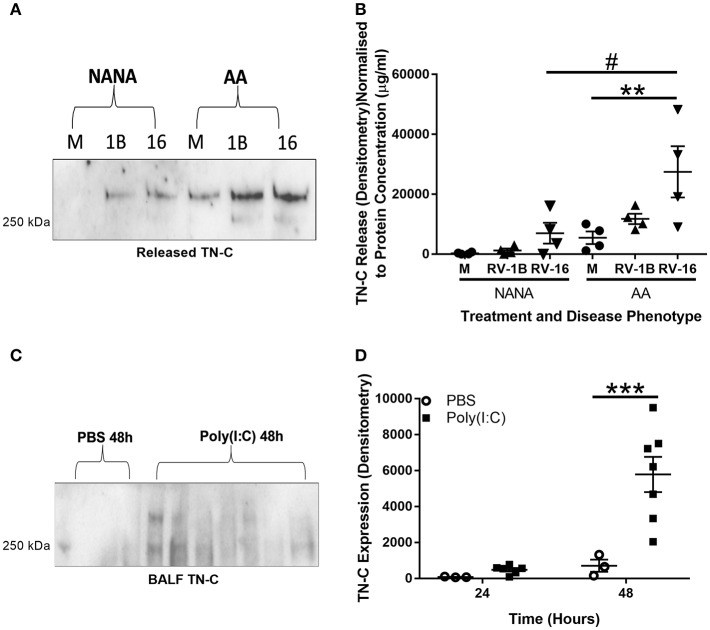
RV infection induces TN-C release *in vitro* and poly(I:C) induces TN-C *in vivo*, with increased release observed in bronchial epithelial cells from people with asthma. **(A,B)** Cell-free supernatants from NANA and AA PBECs, infected with RV-1B and RV-16 for 6 or 24 h, were obtained from the ALLIANCE study. **(A)** Cell-free supernatants were analyzed by western blot using antibodies specific to TN-C (one representative blot shown), with media samples (M), RV-1B (1B), and RV-16 samples (16). **(B)** Densitometry of the large >250 kDa variant was performed in ImageJ and normalized to protein concentration (determined by a bicinchoninic acid assay). Data shown are mean ± SEM with each replicate carried out using an independent PBEC donor (*n* = 4). **(C,D)** Under recovery anesthesia, adult C57BL/6 mice were treated intranasally with 50 μl PBS or 100 μg poly(I:C) in 50 μl PBS for up to 48 h. The mice were then sacrificed, and BALF was collected by washing the lungs with 3 ml of PBS. **(C)** 150 μl of mouse BALF was TCA precipitated to a final volume of 20 μl and the presence of TN-C at 24 and 48 h was analyzed by western blot (48 h blot shown). **(D)** Densitometry of the small ~250 kDa variant was then performed using ImageJ software and normalized to neutrophil cell count. Data shown are mean ± SEM from a single experiment, with each point a separate mouse (3 mice for PBS treatment and 7 mice for poly(I:C) treatment). Significant differences in TN-C secretion are indicated by ^#^*p* < 0.05; **p < 0.01; ***p < 0.001; analyzed by two way ANOVA with Tukey's *post-hoc* test.

TN-C is a protein that, once released, can have a pro-inflammatory function upon interaction with cells such as macrophages (14). Therefore, we first investigated whether epithelial cell TN-C release occurred in response to RV. PBECs from NANA (healthy non-atopic non-asthmatic control) and AA (atopic asthmatic) adult patients were obtained and infected with RV-1B (a minor group RV) and RV-16 (a major group RV) *in vitro*. Western blotting of cell culture supernatant revealed increased TN-C release upon infection with both RV serotypes. When analyzed by densitometry and normalized to total protein concentration (determined by bicinchoninic acid assay), RV-16 infection produced greater TN-C release in AA PBECs compared to the AA media control and to the NANA RV-16 treated samples (Figures 1A,B). Due to previous evidence of TN-C KO mice having reduced AHR severity (16), the relationship between poly(I:C) (a TLR3 agonist and viral mimic) stimulation and TN-C release in an *in vivo* murine model was also investigated. C57BL/6 mice were stimulated intranasally with poly(I:C), using PBS as a vehicle control, sacrificed at the indicated times, and BALF collected. Western blotting BALF revealed TN-C levels were increased 48 h post-poly(I:C) stimulation, compared to the PBS treated controls (Figures 1C,D).

### TN-C Expression and Release in Bronchial Epithelial Cells Is Triggered by TLR3, but Not TLR7, Activation

TN-C release in response to RV infection of human bronchial epithelial cells *in vitro* has not, to our knowledge, been previously determined. Thus, we further investigated this pathway, as well as examining the TLRs responsible. TN-C mRNA levels, and protein expression and release, in response to RV, poly(I:C) and gardiquimod (a TLR7 agonist) in the BEAS-2B cell line and PBECs from healthy donors was determined, with TNFα used as a positive control. TN-C mRNA expression was analyzed by qPCR and normalized to GAPDH expression. Cell-associated TN-C expression and TN-C release was assessed by western blotting cell lysates and cell supernatant, respectively, and normalized to β-actin (lysates only). TN-C levels in cell supernatants were further quantified by ELISA.

**Figure 2:**
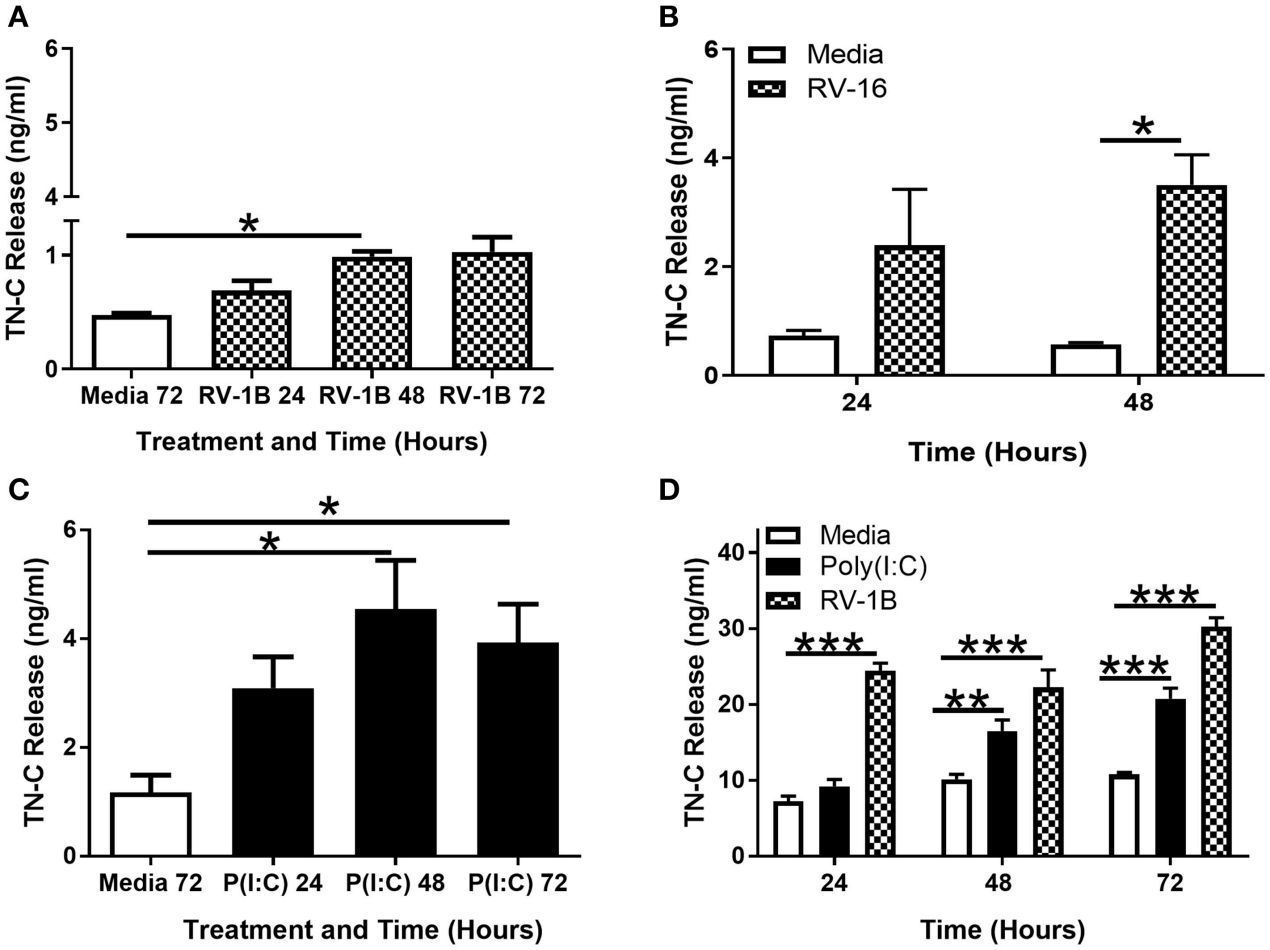
RV induced TN-C release from bronchial epithelial cells is triggered by TLR3 activation and is not RV-serotype specific. PBECs **(A–C)** and BEAS-2B cells **(D)** were treated with poly(I:C) (25 μg/ml), RV-1B (MOI 0.6), or RV-16 (MOI 1.5) for the indicated times. Cell-free supernatants were analyzed by ELISA to measure TN-C release. Data shown are mean ± SEM (*N* = 3–5) with each replicate a separate BEAS-2B cell passage or independent PBEC donor. Significant differences in TN-C release are indicated by *p < 0.05; **p < 0.01; ***p < 0.001; analyzed by Kruskal-Wallis one-way ANOVA with Dunn's *post-hoc* test **(A,C)** or two way repeated measures ANOVA with Tukey's *post-hoc* test **(B,D)**.

TN-C release in PBECs in response to RV-1B (Figure 2A) and RV-16 (Figure 2B) infection was confirmed by TN-C ELISA, with peak TN-C release occurring at 48–72 h post-infection. PBECs also released TN-C upon stimulation with poly(I:C) (Figure 2C), and we confirmed that as expected poly(I:C) induced the release of CXCL8 (Supplementary Figures 1A,C) and CCL5 (Supplementary Figures 1B,D) from both BEAS-2B and PBECs. To confirm the specificity of the TN-C antibody, recombinant TN-C with a histidine-tag was purified and compared to PBEC intracellular associated TN-C by western blot. The purified TN-C displayed the same band pattern as the more complex cellular samples (Supplementary Figure 1E). PBECs did not respond to gardiquimod stimulation (as measured by CXCL8; Supplementary Figure 2A) and TN-C release did not occur (measured by western blot; Supplementary Figure 2B). Gardiquimod activity was confirmed by stimulation of macrophages (Supplementary Figure 2A). Poly(I:C) and RV-1B treatment of BEAS-2B cells also resulted in TN-C release (Figure 2D), with no response to gardiquimod (data not shown). Together these data demonstrate TN-C release is not RV serotype specific and can be promoted by TLR3, but not TLR7 activation in primary human epithelial cells and cell lines.

Upregulation of TN-C mRNA and cell-associated TN-C protein was not observed in PBECs in response to poly(I:C), RV-1B or RV-16 (data not shown), whilst poly(I:C) stimulated TN-C mRNA expression in BEAS-2B cells at 24 h (Supplementary Figure 2C), and cell-associated TN-C protein expression at 24 and 48 h post-stimulation (Supplementary Figure 2D), with levels of expression similar to that induced by TNFα. Basal levels of TN-C mRNA expression (Supplementary Figure 2E) and cell-associated protein (Supplementary Figure 2F) were significantly greater in PBECs compared to BEAS-2B cells. Together these data show a cell-type specific effect of viral infection/poly(I:C) stimulation on TN-C expression, and suggests that the required rate of TN-C transcription needed to facilitate protein release depends on existing intracellular expression.

### TN-C Release Does Not Occur as a Result of Cell Death

The next aim was to investigate the mechanism of TN-C release following viral infection. RV infection of bronchial epithelial cells can promote the induction of apoptosis (27), and it was important to ascertain whether the observed TN-C release is directly promoted by infection or an indirect by-product of RV-induced cytotoxicity.

**Figure 3:**
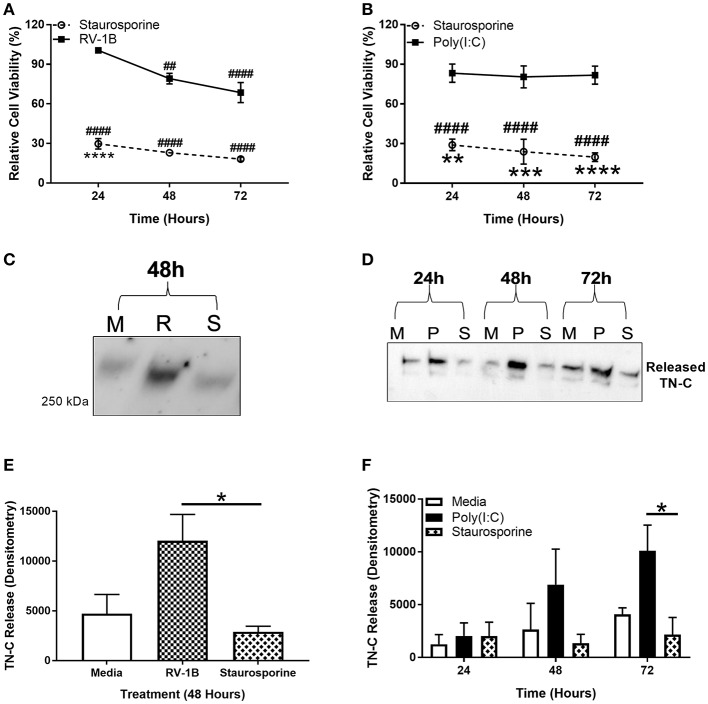
Induction of epithelial cell death is not sufficient to induce PBEC TN-C release. PBECs were grown to confluence and infected with RV-1B (MOI 0.6) or stimulated with poly(I:C) (25 μg/ml) or staurosporine (5 μg/ml) for the indicated times. Metabolic activity was measured by MTT assay in response to RV and staurosporine **(A)** and poly(I:C) and staurosporine **(B)**. The presence of TN-C was analyzed by western blot (M for Media, R for RV-1B, P for poly(I:C), and S for staurosporine; one representative blot shown; **(C,D)**. Densitometry of the large >250 kDa variant in response to RV **(E)** and poly(I:C) **(F)** was performed in ImageJ software. Values are expressed as mean ± SEM (*N* = 3–4) with each replicate representing an independent PBEC donor. Significant differences in cell viability (# compared to media control which was assigned 100% and * compared to poly(I:C)/RV) and TN-C release are indicated by, *p < 0.05; **p < 0.01; ***p < 0.001; ****p < 0.0001; ##*p* < 0.01; ####*p* < 0.0001, analyzed by two way ANOVA with Dunnett's *post-hoc* test. Analysis for MTT assay was performed on raw values.

PBECs were infected with RV, stimulated with poly(I:C), or treated with staurosporine (a promoter of apoptosis) for up to 72 h, and the MTT cell metabolic activity assay performed. Supernatants were collected and TN-C levels were compared by western blot. RV infection resulted in a significant reduction in cell metabolic activity (~30%; Figure 3A) whilst staurosporine treatment caused ~70–80% reduction (Figures 3A,B) compared to the media control. In contrast, poly(I:C) did not affect cell metabolic activity. Importantly, RV infection (Figures 3C,E) and poly(I:C) stimulation (Figures 3D,F) both induced TN-C release from PBECs, whilst staurosporine treatment did not (Figures 3C–F). This indicates that virally induced TN-C release is not associated with significant changes in cell viability and that induction of epithelial cell death is not sufficient to induce TN-C release.

### Poly(I:C) Stimulation of BEAS-2B Cells Induces sEV-Associated TN-C Release

sEVs are a type of extracellular vesicle (EV) that range from 50 to 200 nm in size and originate from the endosomal pathway or the plasma membrane (28, 29). sEVs can encompass exosomes and smaller microvesicles, and were investigated as a potential mechanism of TN-C release as TN-C association with these vesicles has previously been reported in cancerous colorectal cell lines (29) and sEVs have been implicated in asthma pathogenesis and airway inflammation (30). There is a need for large amounts of sEVs to be isolated for accurate NTA analysis and sEVs also have a short storage time before degradation, thus, BEAS-2B cells were chosen as suitable cells, due to their quicker doubling time and greater density in culture than PBECs.

**Figure 4:**
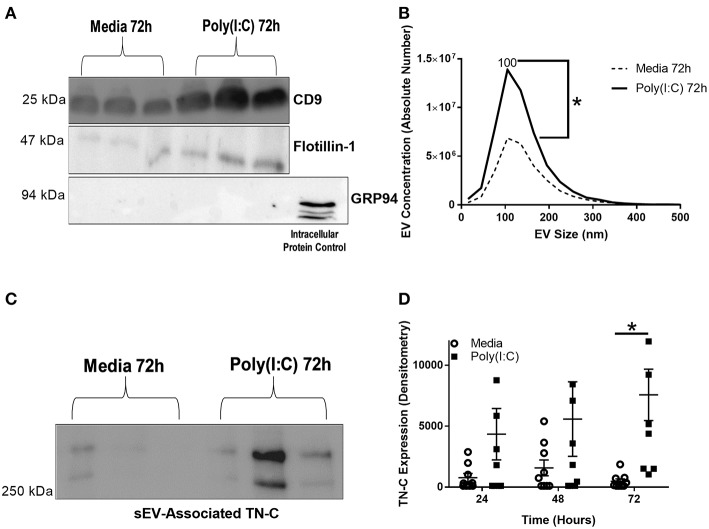
Poly(I:C) stimulation of BEAS-2B cells induces the release of sEVs and sEV-associated TN-C expression. BEAS-2B cells were grown to confluence in EV-depleted media and stimulated with poly(I:C) (25 μg/ml) for the indicated times. sEVs were then isolated by the four spin ultracentrifugation method and re-suspended in 100 μl PBS. **(A)** CD9, flotillin-1 and GRP-94 expression was measured by western blot (one representative blot shown). **(B)** sEV concentration and size was quantified by Nanoparticle Tracking Analysis on ZetaView. **(C)** TN-C expression was measured by western blot (one representative blot shown) and **(D)** TN-C expression was quantified by densitometry using ImageJ software. Values are expressed as mean ± SEM (*N* = 7) with each replicate a different cell passage. Significant differences in sEV release and sEV-associated protein expression are indicated by *p < 0.05, analyzed by Mann-Whitney *U* test **(B)** or two way ANOVA with Tukey's *post-hoc* test **(D)**.

BEAS-2B cells were cultured in EV-depleted media, stimulated with poly(I:C) and supernatant collected for sEV isolation, using a four-step ultracentrifugation method (Supplementary Figure 3A). The sEVs were characterized by western blotting, confirming the presence of sEV enriched-proteins CD9 and flotillin-1, and the absence of intracellular protein control GRP94 in the sEV samples (Figure 4A). The average sEV size, as determined by NTA, was 100 nm and the concentration of sEVs increased at 72 h post-stimulation (Figure 4B). sEV associated TN-C expression was measured by western blot, and increased at 72 h post-stimulation (Figures 4C,D). Analysis of the amount of TN-C left in the supernatant after sEV isolation demonstrated that ~50% of released TN-C is associated with sEVs (Supplementary Figure 3B). Together these data reveal that TN-C release occurs in two formats; soluble TN-C protein in the cell supernatant and TN-C protein associated with sEVs.

### FBG-C and Poly(I:C)-Induced sEVs Induce Cytokine Release in BEAS-2B Cells, but the sEV-Pathway May Not be TN-C-Dependent

This work has demonstrated that RV infection induces the release of soluble TN-C from bronchial epithelial cells, and that TN-C is also associated with poly(I:C)-induced sEV release. As the inflammatory function of TN-C on epithelial cells has not been investigated, we measured the effect of soluble FBG-C on BEAS-2B cells, using macrophages as a control cell type. RV infection has also been hypothesized to induce changes in the sEV miRNA composition, potentially contributing to enhanced airway inflammation and anti-viral activity (31), whilst sEVs from nasal lavage fluid of people with chronic airway inflammation contain altered protein cargo (32). The inflammatory and anti-viral consequence of sEV addition to BEAS-2B cells was therefore investigated, and the role of TN-C in this pathway determined.

**Figure 5:**
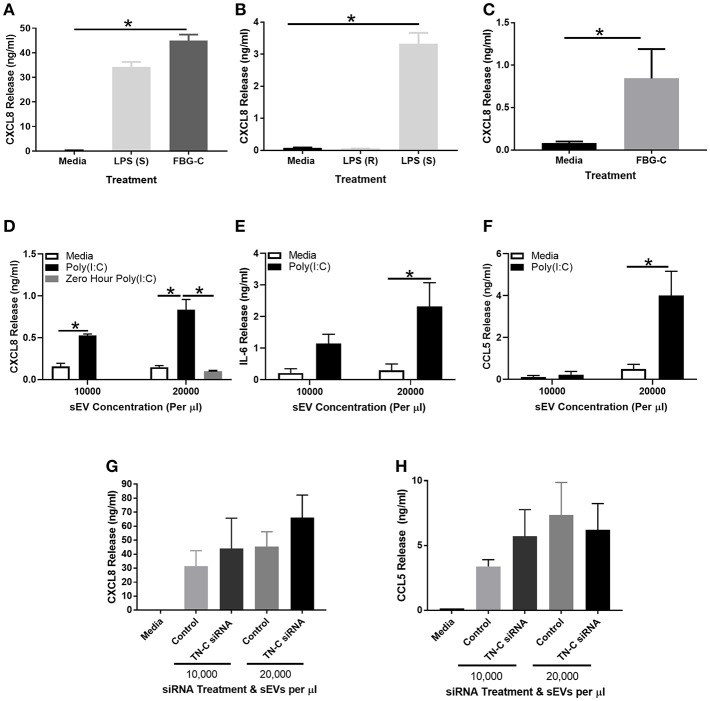
FBG-C induces cytokine release in macrophages and BEAS-2B cells and sEVs isolated from virally-stimulated BEAS-2B cells induce BEAS-2B cell cytokine release which may be independent from TN-C. **(A)** Monocyte derived macrophages were left unstimulated or stimulated with purified recombinant FBG-C (1 μM) or LPS smooth (S) serotype 0111:B4 (10 μg/ml) for 24 h. **(B)** BEAS-2B cells were left unstimulated or stimulated with LPS smooth serotype 0111:B4 (10 μg/ml), LPS rough (R) serotype EH100 (10 μg/ml) or **(C)** FBG-C (1 μM) for 24 h. **(D–F)** BEAS-2B cells were grown to confluence in EV-depleted media, and stimulated with poly(I:C) (25 μg/ml) for 72 h. sEVs were then isolated by the four spin purification method, re-suspended in 100 μl PBS and the concentration measured by NTA. sEVs were added to fresh BEAS-2B cells at the designated concentrations and cell free supernatants were collected at 24 h. **(D)** CXCL8 (with a 0 h poly(I:C) control), **(E)** IL-6 and **(F)** CCL5 were measured by ELISA. **(G,H)** BEAS-2B cells were pre-treated with 100 nM TN-C siRNA or 100 nM control siRNA for 24 h, and then stimulated with poly(I:C) (25 μg/ml) for 72 h. sEVs were isolated by the four-step ultracentrifugation method and TN-C expression was determined by western blot. BEAS-2B cells were then stimulated with the siRNA treated sEVs for 24 h. Cell free supernatants were collected and analyzed for **(G)** CXCL8 and **(H)** CCL5 release by ELISA. Data are expressed as mean ± SEM (*N* = 3–4) with each replicate a different cell passage and separate sEV population. Significant cytokine release is indicated by *p < 0.05, analyzed by Kruskal-Wallis with Dunn's *post-hoc* test **(A,B,G,H)**, Mann-Whitney *U* Test **(C)** or two way ANOVA with Tukey's *post-hoc* test **(D,E,F)**.

FBG-C was purified by Ni^2+^ purification as per (14, 24) and characterized for activity and structure (Supplementary Figures 4A–C). A concentration response curve was generated in BEAS-2B cells, with 1–2 μM FBG-C determined to be sufficient to induce CXCL8 release (Supplementary Figure 4D). MDMs were also stimulated with 1 μM FBG-C, with previous work in our group demonstrating that concentrations between 0.05 and 1 μM were sufficient to induce an inflammatory response (14, 24). FBG-C was added exogenously to macrophages (with an LPS TLR4 positive control) or BEAS-2B cells, cell-free supernatants were collected, and CXCL8 release analyzed by ELISA. FBG-C stimulation resulted in substantial CXCL8 release in macrophages, with the amount released similar to that elicited by LPS stimulation (Figure 5A). Due to contradictory evidence about the ability of bronchial epithelial cells to respond to LPS (and thus respond to TLR4 agonists) (20), BEAS-2B cells were first stimulated with rough LPS serotype EH100 and smooth LPS serotype 0111:B4 and CXCL8 production measured. Smooth LPS, but not rough LPS induced significant cytokine release from BEAS-2B cells (Figure 5B); whilst TN-C-FBG stimulation (1 μM) also elicited CXCL8 release in BEAS-2B cells (Figure 5C).

BEAS-2B cells were next stimulated with poly(I:C) for 72 h and sEVs were isolated as per Supplementary Figure 3A. sEVs were quantified by NTA and added to BEAS-2B cells at 10,000–20,000 sEVs per μl for 24 h, before cell free supernatants were collected for analysis. Addition of unstimulated media control sEVs did not induce any cytokine release from BEAS-2B cells, whilst addition of sEVs from poly(I:C) stimulated cells induced CXCL8 (Figure 5D), IL-6 (Figure 5E), and CCL5 (Figure 5F) release. A 0 h control was used in the CXCL8 experiment, with sEVs isolated instantly after poly(I:C) stimulation. No CXCL8 release was induced, demonstrating a lack of poly(I:C) contamination. To determine what role TN-C played in this response, the experiment was repeated with a 100 nM TN-C siRNA or 100 nM control siRNA pre-treatment step prior to poly(I:C) stimulation. Despite a knockdown efficiency of ~75% (Supplementary Figures 4E,F), there was no difference in poly(I:C) induced CXCL8 (Figure 5G) or CCL5 (Figure 5H) release between the two siRNA groups.

## Discussion

This study identifies two novel pathways that can mediate inflammation in bronchial epithelial cells and macrophages: the release of the ECM protein TN-C in response to RV infection (which is elevated in PBECs from people from asthma), and the generation and release of sEVs in response to TLR3 stimulation by poly(I:C).

**Figure 6:**
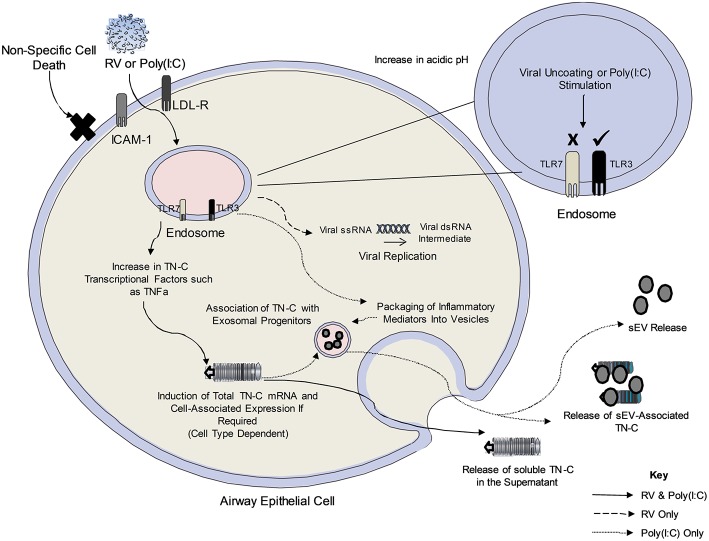
The proposed mechanism of TN-C release following RV infection of bronchial epithelial cells. Upon viral infection, RV is internalized to the early endosome and uncoats. The single stranded RNA exits the endosome (early or late endosome depending on the RV serotype) and forms a temporary double stranded RNA intermediate (recognized by TLR3 in the cytoplasm). It is not yet clear how the endosomal TLR recognizes the cytoplasmic RNA. Poly(I:C) enters the endosome upon addition to cells and stimulates the TLR3 pathway. The amount of RV/Poly(I:C)-dependent cell associated TN-C upregulation may depend on existing basal levels of expression in the cell, with PBECs expressing higher TN-C levels at baseline compared to BEAS-2B cells. Furthermore, RV-induced TN-C release occurs in response to major and minor serotypes of RV, and is not an indirect consequence of cell cytotoxicity, as poly(I:C) induces TN-C release despite no significant cell death. Activation of the TLR3 dependent pathway (and other pathways upon viral infection) induce the release of TNFα and TGFβ, known transcriptional regulators of TN-C through the MAPK/ERK and NF-κB/p65 pathways. It is postulated that this triggers the signaling cascade required for the expression and release of TN-C in PBECs. Black lines denote events determined by experiments with both poly(I:C) and RV, the dashed line indicates events that occur in RV infection and the dotted line indicates events determined by experiments with poly(I:C) only.

The proposed mechanisms of RV-dependent TN-C release and poly(I:C)-dependent sEV release are summarized in Figure 6. Work in this study determined that TN-C release in bronchial epithelial cells is triggered by TLR3, but not TLR7 signaling, with the lack of response to gardiquimod correlating with previous work from our lab (21). TN-C release occurred in response to both minor and major RV serotypes. In contrast, intracellular TN-C was not upregulated in response to RV in PBECs, results which are in keeping with another study, which demonstrated RV infection of primary airway smooth muscle cells did not promote expression of TN-C (33). The amount of RV-dependent TN-C upregulation may depend on basal levels of expression. We theorize that the lack of significant intracellular upregulation of TN-C in PBECs following infection was due to the high basal levels that are present in the cells, and therefore promotion of TN-C expression was not required in order for the protein to be released. This also explains why upregulation was observed in BEAS-2B cells upon stimulation and infection, as these cells express low basal levels of TN-C. As TNFα and TGFβ are known transcriptional regulators of TN-C (34, 35), and are produced in response to RV infection (36), we postulate these cytokines trigger the signaling cascades that promote the release of TN-C from bronchial epithelial cells. This pathway was also confirmed *in vivo*, with nasal administration of poly(I:C) inducing TN-C release in BALF from mice at 48 h post-stimulation. Poly(I:C) was chosen as a relevant stimulus as this model has previously been shown to elicit lung inflammation and impair lung function in mice in a TLR3 dependent manner (37). We confirmed that poly(I:C) produced a robust immune response 48 h post-stimulation (data not shown).

Once released into surrounding environment, soluble TN-C has the ability to induce TLR4 inflammation through the FBG-C domain. BEAS-2B cells in culture have the ability to respond to smooth LPS but not rough LPS [which can be overcome by co-culture with monocytes, which provide the required co-receptors (20)], and our work now reveals that FBG-C can also induce inflammatory cytokine release from bronchial epithelial cells. This draws a parallel with, and builds on, our previous work demonstrating the ability of TN-C to induce inflammatory cytokine release via FBG-C-TLR4 interactions in macrophages and fibroblasts (14). Work in the future will aim to detail exactly how much TN-C is released in the airway following infection, both from AA and NANA samples.

RV infection lasts on average 9–11 days, meaning multiple rounds of infection, viral egress, and reinfection occur (38). Evidence is contradictory about whether asthma suffers are more susceptible to RV infection, however it is clear that RV infection persists longer in people with asthma and they have more severe symptoms (38). The isoforms of TN-C released in response to RV are large (>250 kDa), which are more resistant to matrix metalloproteinase 9 (MMP9) degradation (39) and have a greater capacity to induce inflammation than smaller isoforms (14, 40). In people with asthma, the exaggerated response to RV infection, leading to greater TN-C release, could lead to TN-C persisting in the airway after the clearance of viral infection, incorporating into the ECM and exerting its inflammatory effect locally (15). Furthermore, the release of TN-C could form a positive feedback loop (previously demonstrated in RA), further increasing expression and release of the protein (41). Also, viral infection itself may result in a greater susceptibility to virally induced-TN-C release and the consequential TLR4 dependent inflammation, with respiratory syncytial virus (RSV) having been previously shown to induce the upregulation of TLR4 in bronchial epithelial cells (42).

The work described here also established that poly(I:C) stimulation of BEAS-2B cells induced sEV release and provided novel information that TN-C is associated with these vesicles. Results from this study demonstrate that ~50% of the released TN-C was associated with sEVs, whilst the other 50% was present in the supernatant. This study establishes sEVs as an inflammatory pathway of importance in the airway, with initial results indicating that sEVs exert an inflammatory effect in a TN-C-independent manner. However, as 100% of TN-C knockdown was not achieved, further investigation is required in order to discount TN-C from having a role in TLR3-induced sEV-dependent inflammation. The results in this manuscript are consistent with a recent study that demonstrated that RSV-induced sEVs promote inflammatory cytokine release in an alveolar epithelial cell line through IP-10, CCL2, and CXCL10 release (43). Our work also highlights for the first time that virally-stimulated sEVs promote an anti-viral CCL5 response in surrounding bronchial epithelial cells after infection. sEVs can also “travel” quite large distances of at least several cell diameters (44), and thus will induce a more widespread immune response to RV infection than soluble TN-C.

The revelation that RV infection and poly(I:C) stimulation can induce inflammatory TN-C and/or sEV release is of importance in the context of virally-induced asthma exacerbations. TN-C release was increased in the PBECs of AA subjects in response to RV, potentially providing evidence of a mechanism for the increased expression of the protein in the basement membrane of people with asthma (13, 15). As detailed above, RV infections persist longer in people with asthma (38) and this, alongside increased basal cell-associated expression of TN-C in people with asthma (15), may result in the greater release of the protein upon infection, promoting a chronic inflammatory response and contributing toward the development of AHR. Furthermore, sEVs isolated from the BALF of people with asthma have an increased inflammatory miRNA profile which can contribute toward pathogenesis (45), and thus RV infection may shift the imbalance of sEV miRNA profile even further.

Now the novel mechanisms of RV-induced TN-C release and poly(I:C) induced sEV release have been established, it will be paramount in the future to further characterize these pathways. We aim to investigate whether RV induces sEVs with a similar inflammatory phenotype to poly(I:C), and whether this is potentiated further in people with asthma. TN-C is a protein that can be targeted therapeutically with monoclonal antibodies that target the TLR4 binding epitope on the FBG-C domain, reducing cytokine release in RA synovial cells (19). Thus, TN-C may be a potential therapeutic target in the future in order to reduce a local inflammatory response to TN-C following RV-dependent release. Furthermore, sEVs are currently used as biomarkers in cancers such as colorectal cancer (46), and as more work unveils the role of sEVs in asthma, these vesicles, and the levels of associated TN-C, should be considered as a potential biomarker for disease severity.

The data presented in this study reveals novel consequences of RV infection and poly(I:C) stimulation of bronchial epithelial cells: the induction of pro-inflammatory TN-C release that can activate local cytokine synthesis in the airway, and the release of sEVs (that contain TN-C) which have the ability to induce an immune response over longer distances. The pathway of TN-C release is also more active in the airway of people with asthma and thus identifies TN-C and sEVs as relevant to the biology of virally induced exacerbations of asthma.

## Data Availability

The raw data supporting the conclusions of this manuscript will be made available by the authors, without undue reservation, to any qualified researcher.

## Author Contributions

JM, ME, IS, KM, and LP contributed to the study design. JM performed all experiments apart from those indicated in this paragraph, analyzed the data, and wrote the manuscript. AS completed the FBG purification and characterization study and donated FBG for the use in the study. EM conducted the mouse experiments and donated mouse BALF for use in the study. ME donated supernatants from the asthmatic cells for use in the study. All the authors assisted in the critical review of the manuscript and approved the final version of the manuscript for submission.

### Conflict of Interest Statement

The authors declare that the research was conducted in the absence of any commercial or financial relationships that could be construed as a potential conflict of interest.

## Abbreviations

AA: Atopic Asthmatic
AHR: Airway Hyperresponsiveness
BALF: Bronchoalveolar Lavage Fluid
CD9: Cluster of Differentiation 9
CXCL8: C-X-C Motif Ligand 8
ECM: Extracellular Matrix Protein
ELISA: Enzyme-Linked Immunosorbent Assay
FNIII: Fibronectin Type III-Like
EGF: Epidermal Growth Factor-Like
EV: Extracellular Vesicle
FBG-C: Fibrinogen Globe-Like
GRP94: Glucose Regulated Protein 94
RV: Human Rhinovirus
IRF: Interferon Regulatory Factor
LAL: Limulus Amebocyte Lysate
lEV: Large Extracellular Vesicle
LPS: Lipopolysaccharide
MAPK: Mitogen Activated Protein Kinase
MMP9: Matrix Metalloproteinase 9
MDA5: Melanoma Differentiation-Associated Protein 5
MOI: Multiplicity of Infection
MTT: 3-(4,5-Dimethylthiazol-2yl)-2,5-Diphenyltetrazolium Bromide
NADPH: Nicotinamide Adenine Dinucleotide Phosphate
NANA: Non-Atopic Non-Asthmatic
PBEC: Primary Bronchial Epithelial Cell
PBMC: Peripheral Blood Mononuclear Cell
Poly(I:C): Polyinosinic:Polycytidylic Acid
RA: Rheumatoid Arthritis
RIG-1: Retinoic Acid-Inducible Gene I
RLR: RIG-I-Like Receptor
RSV: Respiratory Syncytial Virus
SNP: Single Nucleotide Polymorphisms
sEV: Small Extracellular Vesicle
TA: Tenascin-Assembly
TCA: Trichloroacetic Acid
TGFβ: Transforming Growth Factor beta
TLR: Toll-Like Receptor
TN-C: Tenascin-C
TNFα: Tumor Necrosis Factor alpha.

## References

[1] O'Byrne PM Inman MD Airway hyperresponsiveness. Chest. (2003) 123:411S−6. 10.1378/chest.123.3_suppl.411S 12629006

[2] Lommatzsch M Severe asthma: definition, diagnosis and treatment. Allergologie. (2016) 39:206–7. 10.5414/ALX01853e PMC4357024 25585581

[3] Aikawa T Shimura S Sasaki H Ebina M Takishima T. Marked goblet cell hyperplasia with mucus accumulation in the airways of patients who died of severe acute asthma attack. Chest. (1992) 101:916–21. 10.1378/chest.101.4.916 1555462

[4] Garbino J Gerbase MW Wunderli W Kolarova L Nicod LP Rochat T. Respiratory viruses and severe lower respiratory tract complications in hospitalized patients. Chest. (2004) 125:1033–9. 10.1378/chest.125.3.1033 15006965

[5] McIntyre CL Knowles NJ Simmonds P. Proposals for the classification of human rhinovirus species A, B and C into genotypically assigned types. J Gen Virol. (2013) 94:1791–806. 10.1099/vir.0.053686-0 23677786 PMC3749525

[6] Schuler BA Schreiber MT Li L Mokry M Kingdon ML Raugi DN. Major and minor group rhinoviruses elicit differential signaling and cytokine responses as a function of receptor-mediated signal transduction. PLoS ONE. (2014) 9:e93897. 10.1371/journal.pone.0093897 24736642 PMC3988043

[7] Bochkov YA Watters K Ashraf S Griggs TF Devries MK Jackson DJ. Cadherin-related family member 3, a childhood asthma susceptibility gene product, mediates rhinovirus C binding and replication. Proc Natl Acad Sci USA. (2015) 112:5485–90. 10.1073/pnas.1421178112 25848009 PMC4418890

[8] Slater L Bartlett NW Haas JJ Zhu J Message SD Walton RP. Co-ordinated role of TLR3, RIG-I and MDA5 in the innate response to rhinovirus in bronchial epithelium. PLoS Pathog. (2010) 6:e1001178. 10.1371/journal.ppat.1001178 21079690 PMC2973831

[9] Triantafilou K Vakakis E Richer EAJ Evans GL Villiers JP Triantafilou M. Human rhinovirus recognition in non-immune cells is mediated by Toll-like receptors and MDA-5, which trigger a synergetic pro-inflammatory immune response. Virulence. (2011) 2:22–9. 10.4161/viru.2.1.13807 21224721 PMC3073236

[10] Manley GCA Stokes CA Marsh EK Sabroe I Parker LC DUSP10 negatively regulates the inflammatory response to rhinovirus through IL-1beta signalling. J Virol. (2018) 93:e01659-18. 10.1128/JVI.01659-18 PMC6321923 30333178

[11] Stack J Doyle SL Connolly DJ Reinert LS O'Keeffe KM McLoughlin RM. TRAM is required for TLR2 endosomal signaling to type I IFN induction. J Immunol. (2014) 193:6090–102. 10.4049/jimmunol.1401605 25385819 PMC4258402

[12] Gern JE. How rhinovirus infections cause exacerbations of asthma. Clin Exp Allergy. (2015) 45:32–42. 10.1111/cea.12428 25270551

[13] Proud D Turner RB Winther B Wiehler S Tiesman JP Reichling TD. Gene expression profiles during *in vivo* human rhinovirus infection insights into the host response. Am J Respir Crit Care Med. (2008) 178:962–8. 10.1164/rccm.200805-670OC 18658112

[14] Midwood K Sacre S Piccinini AM Inglis J Trebaul A Chan E. Tenascin-C is an endogenous activator of Toll-like receptor 4 that is essential for maintaining inflammation in arthritic joint disease. Nat Med. (2009) 15:774–80. 10.1038/nm.1987 19561617

[15] Laitinen A Altraja A Kampe M Linden M Virtanen I Laitinen LA. Tenascin is increased in airway basement membrane of asthmatics and decreased by an inhaled steroid. Am J Respir Crit Care Med. (1997) 156:951–8. 10.1164/ajrccm.156.3.9610084 9310019

[16] Nakahara H Gabazza EC Fujimoto H Nishii Y D'Alessandro-Gabazza CN Bruno NE. Deficiency of tenascin C attenuates allergen-induced bronchial asthma in the mouse. Eur J Immunol. (2006) 36:3334–45. 10.1002/eji.200636271 17125141

[17] Matsuda A Hirota T Akahoshi M Shimizu M Tamari M Miyatake A. Coding SNP in tenascin-C Fn-III-D domain associates with adult asthma. Hum Mol Genet. (2005) 14:2779–86. 10.1093/hmg/ddi311 16115819

[18] Marzeda AM Midwood KS. Internal Affairs: tenascin-C as a clinically relevant, endogenous driver of innate immunity. J Histochem Cytochem. (2018) 66:289–304. 10.1369/0022155418757443 29385356 PMC5958381

[19] Aungier SR Cartwright AJ Schwenzer A Marshall JL Dyson MR Slavny PK. Targeting early changes in the synovial microenvironment: a new class of immunomodulatory therapy? Ann Rheum Dis. (2019) 78:186–91. 10.1136/annrheumdis-2018-214294 30552174 PMC6352652

[20] Stokes CA Ismail S Dick EP Bennett JA Johnston SL Edwards MR. Role of Interleukin-1 and MyD88-dependent signaling in rhinovirus infection. J Virol. (2011) 85:7912–21. 10.1128/JVI.02649-10 21593174 PMC3147909

[21] Parker LC Prestwich EC Ward JR Smythe E Berry A Triantafilou M. A phosphatidylserine species inhibits a range of TLR- but not IL-1 beta-induced inflammatory responses by disruption of membrane microdomains. J Immunol. (2008) 181:5606–17. 10.4049/jimmunol.181.8.5606 18832719 PMC2574035

[22] Stokes CA Kaur R Edwards MR Mondhe M Robinson D Prestwich EC. Human rhinovirus-induced inflammatory responses are inhibited by phosphatidylserine containing liposomes. Mucosal Immunol. (2016) 9:1303–16. 10.1038/mi.2015.137 26906404 PMC4883656

[23] Dhariwal J Cameron A Trujillo-Torralbo MB del Rosario A Bakhsoliani E Paulsen M. Mucosal type 2 innate lymphoid cells are a key component of the allergic response to aeroallergens. Am J Respir Crit Care Med. (2017) 195:1586–96. 10.1164/rccm.201609-1846OC 28085492 PMC5476911

[24] Zuliani-Alvarez L Marzeda AM Deligne C Schwenzer A McCann FE Marsden BD. Mapping tenascin-C interaction with toll-like receptor 4 reveals a new subset of endogenous inflammatory triggers. Nat Commun. (2017) 8:1595. 10.1038/s41467-017-01718-7 29150600 PMC5693923

[25] Dockrell DH Marriott HM Prince LR Ridger VC Ince PG Hellewell PG. Alveolar macrophage apoptosis contributes to pneumococcal clearance in a resolving model of pulmonary infection. J Immunol. (2003) 171:5380–8. 10.4049/jimmunol.171.10.5380 14607941

[26] To WS Aungier SR Cartwright AJ Ito K Midwood KS. Potent anti-inflammatory effects of the narrow spectrum kinase inhibitor RV1088 on rheumatoid arthritis synovial membrane cells. Br J Pharmacol. (2015) 172:3805–16. 10.1111/bph.13170 25891413 PMC4523337

[27] Deszcz L Gaudernak E Kuechler E Seipelt J. Apoptotic events induced by human rhinovirus infection. J Gen Virol. (2005) 86:1379–89. 10.1099/vir.0.80754-0 15831950

[28] Thery C Witwer KW Aikawa E Alcaraz MJ Anderson JD Andriantsitohaina R Minimal information for studies of extracellular vesicles 2018 (MISEV2018): a position statement of the International Society for Extracellular Vesicles and update of the MISEV2014 guidelines. J Extracell Vesicles. (2019) 8:1535750 10.1080/20013078.2018.1535750 PMC6322352 30637094

[29] Ji H Greening DW Barnes TW Lim JW Tauro BJ Rai A. Proteome profiling of exosomes derived from human primary and metastatic colorectal cancer cells reveal differential expression of key metastatic factors and signal transduction components. Proteomics. (2013) 13:1672–86. 10.1002/pmic.201200562 23585443

[30] Paredes PT Esser J Admyre C Nord M Rahman QK Lukic A Bronchoalveolar lavage fluid exosomes contribute to cytokine and leukotriene production in allergic asthma. Allergy. (2012) 67:911–9. 10.1111/j.1398-9995.2012.02835.x 22620679

[31] Gutierrez MJ Gomez JL Perez GF Pancham K Val S Pillai DK. Airway secretory microRNAome changes during rhinovirus infection in early childhood. PLoS ONE. (2016) 11:e0162244. 10.1371/journal.pone.0162244 27643599 PMC5028059

[32] Lasser C O'Neil SE Shelke GV Sihlbom C Hansson SF Gho YS. Exosomes in the nose induce immune cell trafficking and harbour an altered protein cargo in chronic airway inflammation. J Transl Med. (2016) 14:181. 10.1186/s12967-016-0927-4 27320496 PMC4913423

[33] Kuo C Lim S King NJC Johnston SL Burgess JK Black JL. Rhinovirus infection induces extracellular matrix protein deposition in asthmatic and nonasthmatic airway smooth muscle cells. Am J Physiol Lung Cell Mol Physiol. (2011) 300:L951–7. 10.1152/ajplung.00411.2010 21460120

[34] Jinnin M Ihn H Asano Y Yamane K Trojanowska M Tamaki K. Tenascin-C upregulation by transforming growth factor-beta in human dermal broblasts involves Smad3, Sp1, and Ets1. Oncogene. (2004) 23:1656–67. 10.1038/sj.onc.1207064 15001984

[35] Nakamura Y Esnault S Maeda T Kelly EAB Malter JS Jarjour NN. Ets-1 regulates TNF-alpha-induced matrix metalloproteinase-9 and tenascin expression in primary bronchial fibroblasts. J Immunol. (2004) 172:1945–52. 10.4049/jimmunol.172.3.1945 14734780

[36] Kim J Sanders SP Siekierski ES Casolaro V Proud D. Role of NF-kappa B in cytokine production induced from human airway epithelial cells by rhinovirus infection. J Immunol. (2000) 165:3384–92. 10.4049/jimmunol.165.6.3384 10975857

[37] Stowell NC Seideman J Raymond HA Smalley KA Lamb RJ Egenolf DD. Long-term activation of TLR3 by poly(I:C) induces inflammation and impairs lung function in mice. Respir Res. (2009) 10:43. 10.1186/1465-9921-10-43 19486528 PMC2694181

[38] Corne JM Marshall C Smith S Schreiber J Sanderson G Holgate ST. Frequency, severity, and duration of rhinovirus infections in asthmatic and non-asthmatic individuals: a longitudinal cohort study. Lancet. (2002) 359:831–4. 10.1016/S0140-6736(02)07953-9 11897281

[39] Siri A Knauper V Veirana N Caocci F Murphy G Zardi L. Different susceptibility of small and large human tenascin-C isoforms to degradation by matrix metalloproteinases. J Biol Chem. (1995) 270:8650–4. 10.1074/jbc.270.15.8650 7536739

[40] Hasegawa M Nakoshi Y Muraki M Sudo A Kinoshita N Yoshida T. Expression of large tenascin-C splice variants in synovial fluid of patients with rheumatoid arthritis. J Orthopaed Res. (2007) 25:563–8. 10.1002/jor.20366 17262825

[41] Goh FG Piccinini AM Krausgruber T Udalova IA Midwood KS. Transcriptional regulation of the endogenous danger signal tenascin-c: a novel autocrine loop in inflammation. J Immunol. (2010) 184:2655–62. 10.4049/jimmunol.0903359 20107185

[42] Monick MM Yarovinsky TO Powers LS Butler NS Carter AB Gudmundsson G. Respiratory syncytial virus up-regulates TLR4 and sensitizes airway epithelial cells to endotoxin. J Biol Chem. (2003) 278:53035–44. 10.1074/jbc.M308093200 14565959

[43] Chahar HS Corsello T Kudlicki AS Komaravelli N Casola A. Respiratory syncytial virus infection changes cargo composition of exosome released from airway epithelial cells. Scie Rep. (2018) 8:387. 10.1038/s41598-017-18672-5 29321591 PMC5762922

[44] Panakova D Sprong H Marois E Thiele C Eaton S. Lipoprotein particles are required for Hedgehog and Wingless signalling. Nature. (2005) 435:58–65. 10.1038/nature03504 15875013

[45] Levanen B Bhakta NR Paredes PT Barbeau R Hiltbrunner S Pollack JL. Altered microRNA profiles in bronchoalveolar lavage fluid exosomes in asthmatic patients. J Allergy Clin Immunol. (2013) 131:894–903. 10.1016/j.jaci.2012.11.039 23333113 PMC4013392

[46] Hon KW Abu N Ab Mutalib NS Jamal R. Exosomes as potential biomarkers and targeted therapy in colorectal cancer: a mini-review. Front Pharmacol. (2017) 8:583. 10.3389/fphar.2017.00583 28894420 PMC5581359

