# Supplementary material for: Airway Epithelial Cells Generate Pro-inflammatory Tenascin-C and Small Extracellular Vesicles in Response to TLR3 Stimuli and Rhinovirus Infection
Source: Front Immunol. 2019 Aug 21;10:1987. doi: 10.3389/fimmu.2019.01987 (PMC6712508; doi:10.3389/fimmu.2019.01987)
Supplement: Supplementary file 2 [file Data_Sheet_2.PDF]

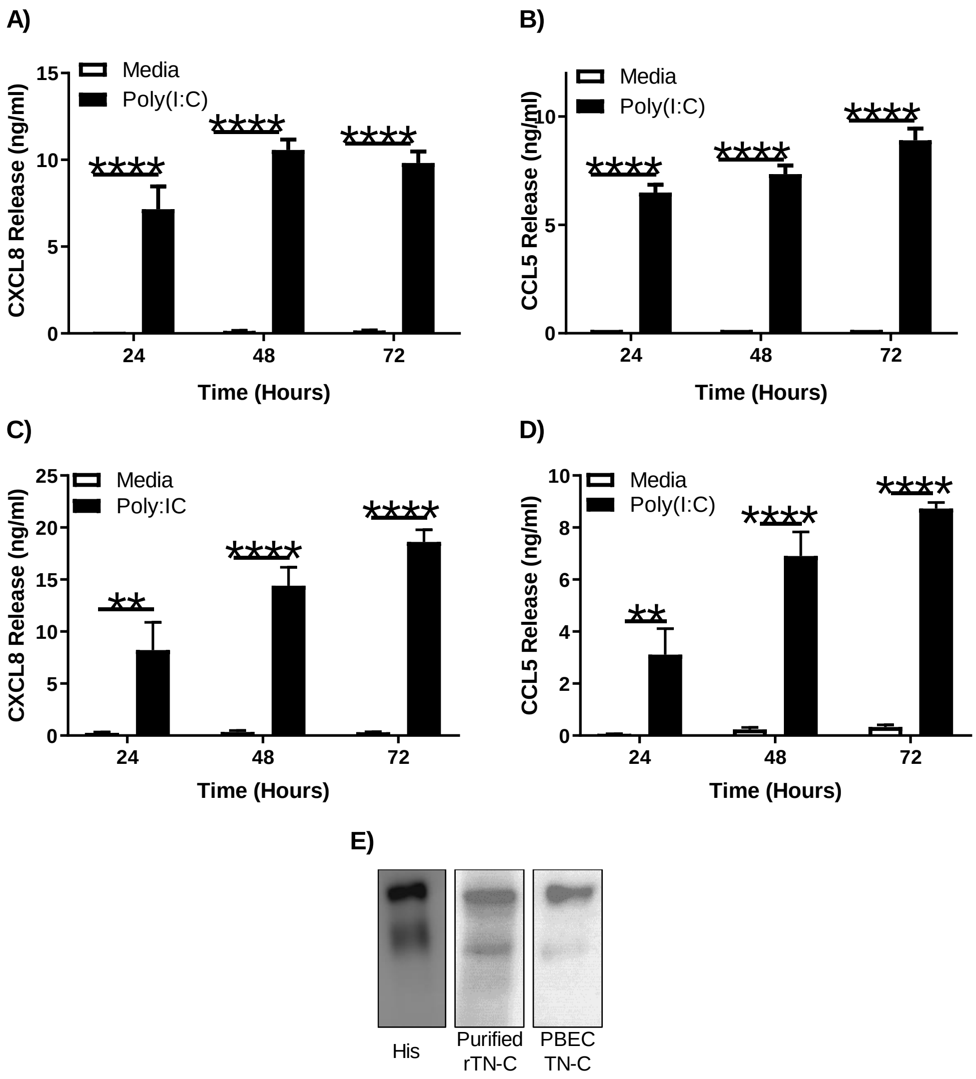

Supplemental Figure 1. BEAS-2B Cells and PBECs respond to poly(I:C) stimulation and MAB1908 antibody is specific to TN-C

BEAS-2B cells **(A-B)** and PBECs **(C-D)** were left unstimulated or treated with poly(I:C) (25 µg/ml) for the indicated times. Cell free supernatants were collected and CXCL8 **(A & C)** and CCL5 **(B & D)** ELISAs performed. **(E)** Specificity of MAB1908 TN-C (N-terminal) antibody was confirmed, with comparison of purified recombinant (r) TN-C (with his-tag) and PBEC cell associated TN-C. Data shown are mean ± SEM (N=3) with each replicate a separate BEAS-2B passage or independent PBEC donor. Significant differences in TN-C expression and release or CXCL8 production are indicated by * $p<0.05$; ** $p<0.01$; **** $p<0.0001$, analysed by two way repeated measures ANOVA with Tukey's post-hoc test.

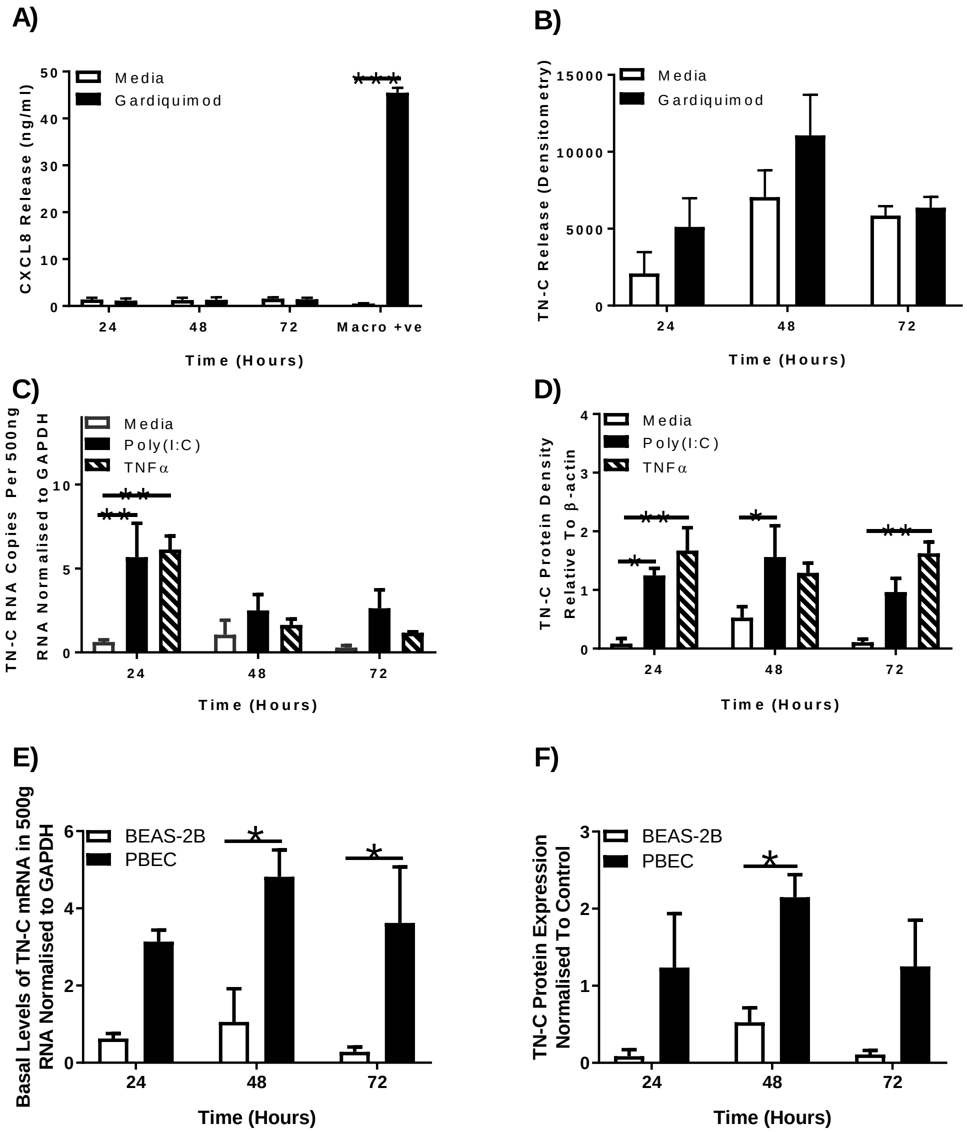

Supplemental Figure 2. PBECs do not respond to TLR7 stimulation and basal levels of TN-C mRNA and protein are greater in PBECs compared to BEAS-2B cells

(A, B) PBECs and macrophages were left unstimulated or treated with gardiquimod (10 µg/ml), **(C, D)** BEAS-2B cells were left unstimulated or treated with poly(I:C) (25 µg/ml) or TNFα (100 ng/ml) and **(E, F)** BEAS-2B and PBECs were left unstimulated for the indicated times. **(A)** Cell free supernatants were collected and CXCL8 ELISA performed, with a macrophage 24 hour positive control (macro +ve). **(B, D, F)** Whole-cell lysates were analysed by western blot using antibodies specific to TN-C (N-Terminal) or ß-actin, and densitometry of the large >250 KDa band was performed in ImageJ software, with cell-associated TN-C normalised to ß-actin. **(C, E)** RNA was extracted from lysates and total TN-C mRNA expression (FBG domain) was measured using TaqMan quantitative real time PCR, with data presented as the total RNA copies normalised to GAPDH control. Data shown are mean ± SEM (N=3) with each replicate a separate BEAS-2B passage or independent PBEC donor. Significant differences in TN-C expression and release or CXCL8 production are indicated by * $p<0.05$; ** $p<0.01$, analysed by two way repeated measures ANOVA with Tukey's post-hoc test.

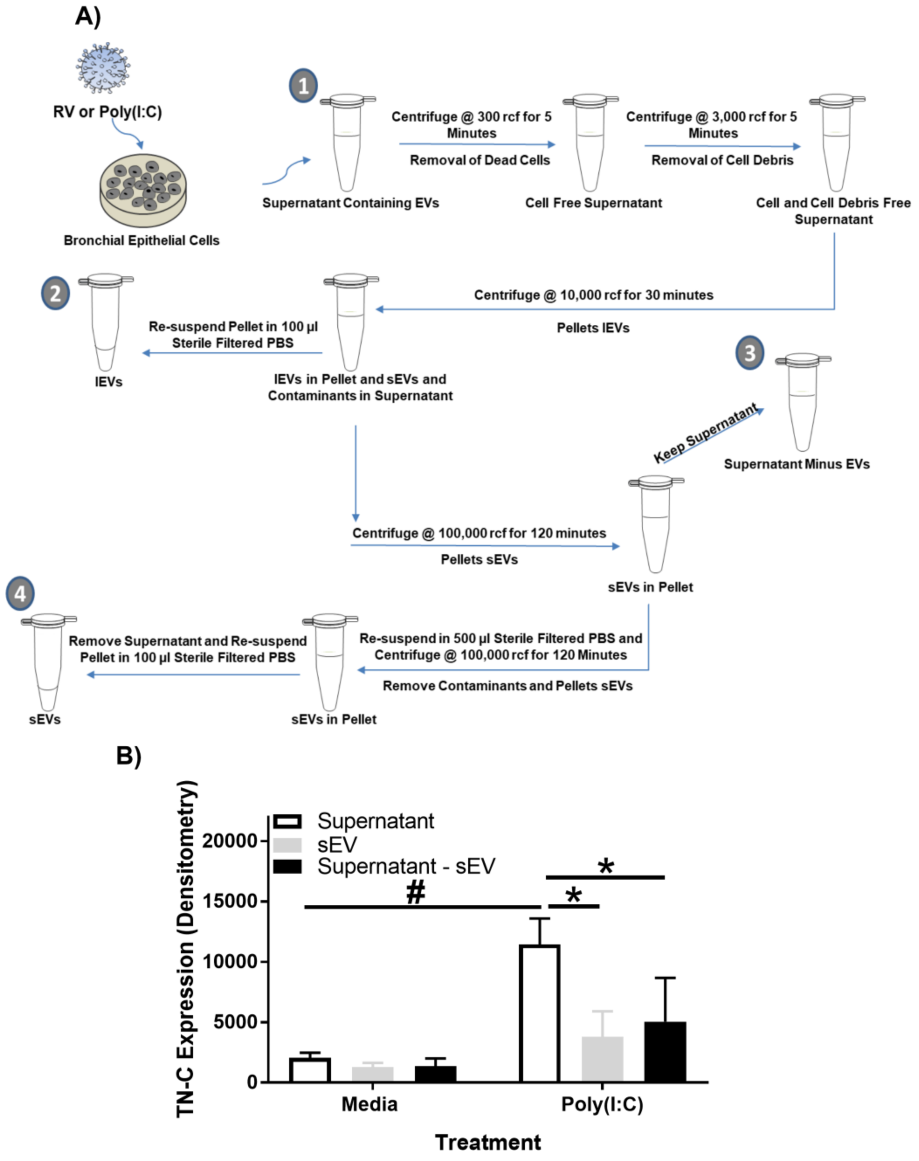

Supplemental Figure 3. The isolation of sEVs from BEAS-2B cell supernatant

(A) Following poly(I:C) stimulation or RV infection, the supernatant (fraction 1) was collected and both large extracellular vesicles (lEVs) and sEVs were then isolated by ultracentrifugation. First, the supernatant was centrifuged at 300 rcf for 5 minutes at 4°C in order to remove dead cells and the supernatant was then collected. Next, the supernatant was centrifuged at 3,000 rcf for 5 minutes at 4°C in order to remove cell debris. The supernatant was then centrifuged at 10,000 rcf for 30 minutes at 4°C in order to isolate lEVs, re-suspended in 100 µl sterile filtered PBS and stored at 4°C (fraction 2). The supernatant was next centrifuged in an ultracentrifuge at 120,000 rcf for 2 hours at 4°C to pellet sEVs, and the supernatant was kept and stored at 4°C (fraction 3). sEVs were then washed in 500 ml of sterile filtered PBS and centrifuged at 100,000 rcf for 2 hours, before the supernatant was removed and the sEVs re-suspended in 100 µl sterile filtered PBS (fraction 4). The sEVs were then stored at 4°C. **(B)** TN-C expression in supernatant, sEV and supernatant minus sEV fractions were measured by western blot and TN-C expression quantified by densitometry using ImageJ software. Values are expressed as mean ± SEM (N=3) with each replicate a different cell passage. Significance differences in TN-C expression are indicated by * $p<0.05$ (* between stimulation groups and # across stimulation groups), analysed by two way ANOVA with Dunnett's test.

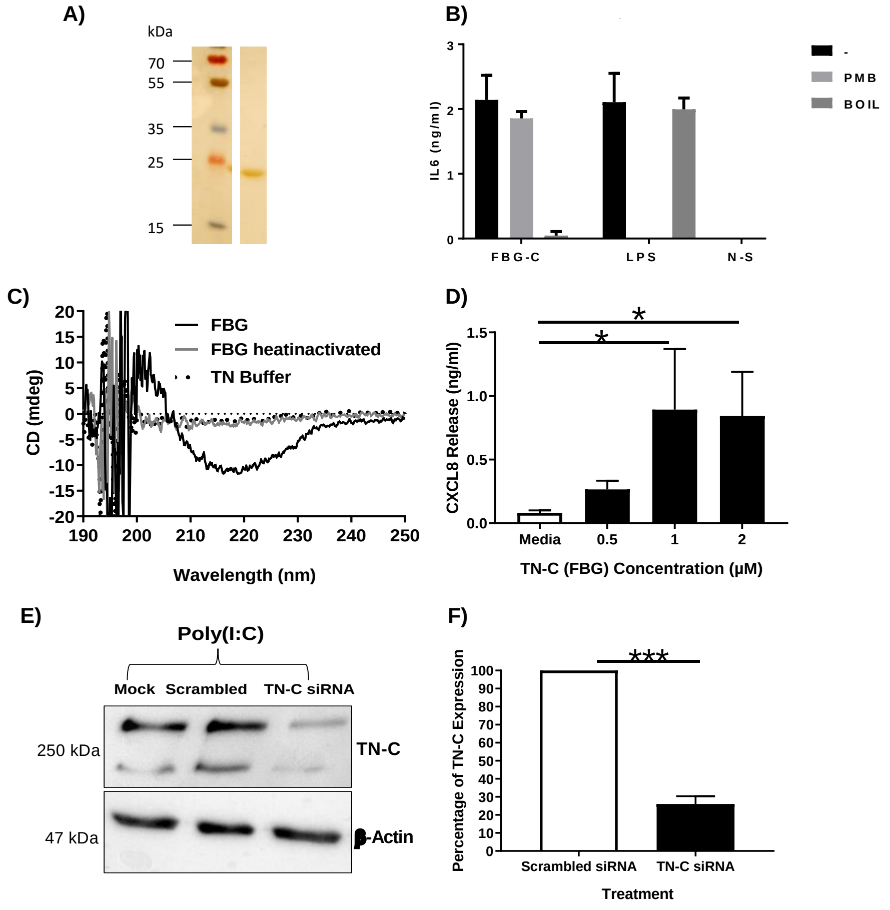

Supplemental Figure 4. Characterisation of recombinant purified FBG-C, FBG-C induces CXCL8 release in BEAS-2B cells above 1µM, and siRNA transfection reduced sEV-associated TN-C in BEAS-2B cells following poly(I:C) stimulation

(A) Protein purity was verified by silver staining of 1 µg of FBG-C. **(B)** Primary human macrophages were left unstimulated (n-s), stimulated with FBG-C (1 µM), incubated for 30 min with polymyxin B (PMB) or boiled for 30 min. IL-6 synthesis was measured by ELISA. **(C)** Circular dichroism (CD) spectra in the far UV region shows folding of FBG-C (black line). FBG-C boiled for 15 minutes unfolds and does not exhibit a characteristic peak at 220 nm (dotted line).The buffer control signal is shown as a grey line. Contamination with endotoxins was measured with the PyroGeneTM Recombinant Factor C Endpoint Fluorescent Assay and no significant contamination was detected (3.4 pg/ml in 1 µM sample). **(D)** BEAS-2B cells were left unstimulated or stimulated with 0.5µM, 1.0µM or 2.0µM recombinant FBG-C for 24 hours, supernatant was collected and CXCL8 ELISA performed. BEAS-2B were pre-treated with 100 nM TN-C siRNA, 100 nM scrambled siRNA or water (mock control) with lipofectamine for 4 hours, before being replaced with fresh media for 24 hours. The cells were then left unstimulated or stimulated with poly(I:C) (25 µg/ml) for 72 hours. **(ED)** sEV-associated TN-C and b-actin expression were analysed by western blot (one representative blot for each shown) and **(FE)** quantification of the large >250 kDa band was performed using ImageJ software. Values are expressed as mean ± SEM (N=3) with each replicate representing a separate cell passage, except for the IL-6 data, which is displayed as mean + SD from one representative donor. Significant differences in TN-C expression indicated by ** $p<0.05$, analysed by Two Way ANOVA with Dunnett's post-hoc test. Analysis was performed on absolute densitometrical values prior to normalisation.
