# Supplementary material for: Airway Epithelial Cells Generate Pro-inflammatory Tenascin-C and Small Extracellular Vesicles in Response to TLR3 Stimuli and Rhinovirus Infection
Source: Front Immunol. 2019 Aug 21;10:1987. doi: 10.3389/fimmu.2019.01987 (PMC6712508; doi:10.3389/fimmu.2019.01987)
Supplement: Supplementary file 3 [file Data_Sheet_3.PDF]

Supplementary Methods

Gardiquimod and TNFα cell stimulation

PBECs or MDMs were seeded in 12 plates, grown to 80% confluency and placed in supplement free media overnight. Cells were stimulated with 10 μg/ml gardiquimod (Invitrogen, Paisley, UK) or 100 ng/ml TNFα (Invitrogen). Cell free supernatants, mRNA and/or protein lysates were then harvested and stored appropriately.

RT-qPCR

RNA was prepared following stimulation / infection and converted to cDNA as described [1]. qPCR was carried out using TaqMan DNA polymerase (Applied Biosystems, Paisley, UK) / GoTaq DNA polymerase (Promega, Southampton, UK) and primer-probes specific to TN-C (FBG-C domain, Hs01115663_m1, Applied Biosystems) and GAPDH (Hs02758991_g1, Applied Biosystems). Target genes were quantified against a standard curve of plasmids containing known copy numbers and TN-C expression was normalised to GAPDH.

sEV isolation

Prior to cell culture, fetal calf serum (FCS; PromoCell, Heidelberg, Germany) was spun in an ultracentrifuge at 120,000 rcf for 18 hours at 4°C (Sorvall Discovery M150 SE ultracentrifuge). This was then added to cell media to create EV-depleted media. Cells were stimulated with poly(I:C) and sEVs were isolated by a four step ultracentrifugation method with a PBS wash step, as described in Figure S2A.

FBG-C purification and characterisation

FBG-C domain with a his-tag was synthesised, purified and characterised as described [2, 3]. To summarise, proteins were expressed in *E. coli* BL21 (DE3) or Rosetta / HEK-293 cells and purified using Ni^{2+} chromatography (Bio-Rad, California, USA). Proteins were characterised by silver staining. Protein secondary structure was determined using the J-815 Circular Dichroism Spectrometer (Jasco, Essex, UK) and analysed using Spectra Manager™ II software. A limulus amebocyte lysate (LAL) test (Lonza, Basel, Switzerland) was then performed, according to the manufacturer's instructions, to determine the LPS concentration. Endotoxin values measured between 0 and 9 pg ml^{-1} are deemed acceptable, and preps with a value higher than 10 pg ml^{-1} are discarded. A bicinchoninic acid assay (VWR International, Pennsylvania, USA) was then used to calculate protein concentration as per the manufacturer's instructions.

siRNA transfection

siRNA transfection was performed using TN-C siRNA (L-009298-00-0005; Dharmacon, Colorado, USA) or control siRNA (D-001810-02-05; Dharmacon) using a protocol as previously described [1].

Animal Work

Additional ethics

Mice were checked prior to experiments by competent personal licensees and were deemed to be fit before start of experiments. The breeding colony was maintained as a het x het closed in house colony.

Housing

WT mice were maintained together in pathogen free, open top cages, provided with ecopure flakes 6 sawdust (Datesand, Manchester, UK) as bedding material and paper wool (Datesand) as nest building material. All cages were supplied with a red plastic mouse house (NKP Plastics, Kent, UK) and were provided with forage mix (LBS Biotech, Horley, UK) once a week after cage cleaning. Animals were kept in stable social groups according to treatments, with 1 cage mate and no more than 5 animals per cage. All animals were fed *ad libitum* with irradiated Teklad global 18% protein rodent diet (Envigo, Derby, UK). Mice were provided with tap water filtered to 0.1 microns *ad libitum*. Water was changed 3 times per week. All animals were kept on a 12 h light/dark cycle.

Study design and additional experimental procedures

Animals were age and sex matched prior to randomised, blind treatments by an authorised technician. N numbers in figure legends correspond to single animals. Group sizes were informed from previous experimental procedure [4]. All animals used in procedures were adults between 45 and 166 days old with a mean age of 74.71 days. Animals weighed between 15.4 and 33.2 g, with a mean weight of 23.48 g. All procedures were performed first thing in the morning after the 12-hour dark cycle. Mice were anesthetized using a small animal anaesthesia machine (AW Anaesthesia Services Ltd, Stoke-On-Trent, UK), and were exposed to 4% gaseous isoflurane mixture delivered in O_2 (5 l/min). Mice were placed in a warmed cage and allowed to recover with access to food and water. Mice were checked after treatments every couple of hours following recovery, and animals were weighed and monitored daily for signs of respiratory distress and weight loss of more than 20% starting weight.

References

[1] G.C.A. Manley, C.A. Stokes, E.K. Marsh, I. Sabroe, L.C. Parker, DUSP10 negatively regulates the inflammatory response to rhinovirus through IL-1beta signalling, J Virol (2018).

[2] K. Midwood, S. Sacre, A.M. Piccinini, J. Inglis, A. Trebaul, E. Chan, S. Drexler, N. Sofat, M. Kashiwagi, G. Orend, F. Brennan, B. Foxwell, Tenascin-C is an endogenous activator of Toll-like receptor 4 that is essential for maintaining inflammation in arthritic joint disease, Nature Medicine 15(7) (2009) 774-U11.
[3] L. Zuliani-Alvarez, A.M. Marzeda, C. Deligne, A. Schwenzer, F.E. McCann, B.D. Marsden, A.M. Piccinini, K.S. Midwood, Mapping tenascin-C interaction with toll-like receptor 4 reveals a new subset of endogenous inflammatory triggers, Nature Communications 8 (2017).
[4] D.H. Dockrell, H.M. Marriott, L.R. Prince, V.C. Ridger, P.G. Ince, P.G. Hellewell, M.K. Whyte, Alveolar macrophage apoptosis contributes to pneumococcal clearance in a resolving model of pulmonary infection, J Immunol 171(10) (2003) 5380-8.
